# An Analysis of Medical Care Services for Children With Rare Diseases in the Russian Federation

**DOI:** 10.3389/fphar.2021.754073

**Published:** 2021-11-24

**Authors:** Svetlana Ya. Volgina, Alexey A. Sokolov

**Affiliations:** ^1^ Department of Pediatrics, Kazan State Medical University, Kazan, Russia; ^2^ Department of Anesthesiology and Intensive Care, North-Western State Medical University, Saint Peterburg, Russia

**Keywords:** rare diseases, child health protection, medical genetic service, preferential provision of medicines, Russia

## Abstract

Rare diseases continue to present numerous challenges for the medical field worldwide. Understanding innovative mechanisms of service provision for patients with rare conditions through shared communication across different healthcare systems should be encouraged. This study presents the organization of medical care for people with rare diseases in Russia, while also exploring the epidemiology of both life-threatening and chronic, progressive, rare diseases. Further, the regulation of medical care provision is examined, including the preferential provision of medicines in different Russian regions and potential role of compulsory medical insurance. The principles guiding patient referrals to appropriate specialist centres for rare diseases are outlined, including considering the increased role that public-patient organizations have in developing healthcare systems. In reviewing the specialized resources available for patients with rare diseases, medical genetics services offering diagnostics and counselling are discussed. Additionally, population-level preventive care necessitates significant investment, principally in diagnostic technology and screening programs. As seen elsewhere, these initiatives involve forming reference centres and tertiary-level pediatric departments staffed by multidisciplinary specialists in rare diseases. Numerous challenges are highlighted relating to Russian healthcare systems, including the financing of expensive treatments and ensuring equitable access to medical care for those patients with rare diseases outside of State-subsidized programs. Recommendations are made on creating international registries for knowledge sharing, quality appraisal, newborn screening, diagnostic challenges, available treatments and rehabilitation services. Given the high cost of rare diseases, cost-effective interventions are advisable, particularly developing preventive programs and targeting the most common and severe mutations in patients planning pregnancies.

## Introduction

Rare diseases, although rare individually, collectively affect a significant proportion of the population, corresponding to 263–446 million people worldwide (3.5–5.9%) (Nguengang Wakap et al., 2020). The Council of the European Union has suggested that between 6 and 8% of Europe’s population could be affected by rare diseases during their lifetime (27–36 million people) (Council of the European Union, 2009), while up to 10% of the United States population are afflicted, amounting to approximately 33 million people. (Herder, 2017). In Japan this number is about 12.5 million people. (Richter et al., 2015). Based on the presented data, it is possible to assume that in Russia rare diseases could be prevalent in about 8–11 million people. Currently it is assumed that the number of rare diseases exceeds 10,000 (Haendel et al., 2020). These diseases often present in severe and potentially life-threatening forms with a profound effect on patients’ quality of life, with 69.9% of cases noted in paediatric populations. (Nguengang Wakap et al., 2020).

The peculiarities of rare diseases make this area a major public health challenge. The limited number of patients and the lack of appropriate knowledge and experience make rare diseases a specialised and high cost field in many countries. Huge amounts of money are spent every year on hospitalization of these patients, including emergency care, medications, home care and outpatient visits, dental, palliative and rehabilitative care as well as insurance reimbursement, the allocation of assistive devices and social services (Angelis et al., 2015; Chiu et al., 2018; Friedlander et al., 2019). Today, international cooperation is required in order to promptly share scientific advances, enabling the maximisation and efficient use of resources in the management of rare diseases worldwide.

## Legislative Regulation of Medical Care, Including Preferential Drug Supply to Patients With Rare Diseases

Human life and health are the highest social values proclaimed in the Constitution of the Russian Federation. Legislative regulation of rare diseases started with the adoption of the Federal law from November 21, 2011 No 323-FL (article 44) (Federal Law No, 2021), according to which a rare disease is defined as a condition that affects not more than 10 people per 100 000 population. The concept of a “rare disease medication” was legally introduced in 2014. As of January 1, 2020, the total number of patients on the Federal Register was 21,594.

## Currently in Russia There are Three Lists of Rare Diseases and Two Subsidized Drug Supply Programs for Patients

1) The general list of rare diseases is created on the basis of their prevalence - not more than 10 cases per 100 000 people, regardless of the presence or absence of drug therapy. It includes 272 diseases (List of rare diseases from 5.11, 2020).
2) Federal list of high-cost diseases, including rare diseases (Table 1) (Federal Law No. (2021)).

**TABLE 1:** Federal list of high-cost diseases, including rare diseases.

No	High-cost diseases, including rare diseases	Code^a^
1	Hemophilia	D66, D67, D68.0
2	Cystic fibrosis	E84
3	Growth Hormone Deficiency	E23.0
4	Gaucher disease	E75.2
5	Tumors of the hematopoietic and lymphoid tissues	C92.1, C88.0, C90.0, C82, C83.0, C83.1, C83.3, C83.4, C83.8, C83.9, C85, C91.1
6	Multiple Sclerosis	G35
7	Hemolytic-uremic syndrome	D59.3
8	Systemic-onset juvenile rheumatoid arthritis	M08.2
9	Mucopolysaccharidosis type I	E76.0
10	Mucopolysaccharidosis type II	E76.1
11	Mucopolysaccharidosis type IV	E76.2
12	Unspecified aplastic anemia	D61.9
13	Inherited factor II deficiency (fibrinogen), factor VII deficiency (labile), factor X deficiency (Stewart-Prauer)	D68.2
14	Patients after organ and/or tissue transplantation	Z94.0, Z94.1, Z94.4, Z94.8

a International Statistical Classification of Diseases and Related Health Problems, 10th revision.

The drug supply for these patients falls under the authority of the federal executive body and is carried out at the expense of the budgetary allocations provided by the federal government. The advantages of this program include the centralized purchase of drugs, which in turn enables a reduction in prices and allows a more efficient use of allocated budgets; the possibility to redistribute drugs between subjects; ensuring equal access to the necessary treatment throughout the Russian Federation. This program was developed in 2008 to treat patients with the most financially burdening diseases and has become one of the most successful medication supply and allocation projects ever implemented by the State.

3) The regional list of life-threatening and chronic progressive rare diseases, leading to a reduction in life expectancy of citizens or their disability (hereinafter referred to as “The regional list of rare diseases”), which is approved by the Government of the Russian Federation (Herder, 2017). The list includes 17 diseases (Table 2) (Resolution of the Government of the Russian Federation of April 26, 2012).

**TABLE 2:** The regional list of life-threatening and chronic progressive rare diseases, leading to a reduction in life expectancy of citizens or their disability.

No	Rare diseases	Code^a^
1	Paroxysmal nocturnal hemoglobinuria	D59.5
2	Immune thrombocytopenic purpura (Evans syndrome)	D69.3
3	Complement deficiency	D84.1
4	Central precocious puberty	E22.8
5	Disorders of aromatic amino acid metabolism (classical phenylketonuria, other types of hyperphenylalaninemia)	E70.0, E70.1
6	Tyrosinemia	E70.2
7	Maple syrup urine disease	E71.0
8	Other types of branched-chain amino acid metabolism disorders (isovaleriс acidemia, methylmalonic acidemia, propionic acidemia)	E71.1
9	Fatty acid metabolism disorders	E71.3
10	Homocystinuria	E72.1
11	Glutaric aciduria	E72.3
12	Galactosemia	E74.2
13	Other sphingolipidoses: Fabry disease, Niemann-Pick disease	E75.2
14	Acute intermittent (hepatic) porphyria	E80.2
15	Copper metabolism disorders (Wilson’s disease)	E83.0
16	Osteogenesis imperfecta	Q78.0
17	Pulmonary (arterial) hypertension (idiopathic) (primary)	I27.0

a International Statistical Classification of Diseases and Related Health Problems, 10th revision.

The executive bodies of the regions of the Russian Federation are legally charged with the responsibility of organizing the preferential drug supply for this category of patients. Financial support for this program is provided from the budgets of the regions of the Russian Federation. As time has shown, the high cost of medications to treat rare diseases has been a significant financial burden on the budgets of the regions of the Russian Federation. This has been an obstacle to the fulfillment of the obligations imposed on them to finance the procurement of necessary medicines. There is noted to be a significant regional difference in the level of satisfaction of this group of patients when considering the provision of medications.

In 2021 a Presidential Decree on the creation of a special Fund to support children with 27 rare diseases was issued (Table 3) (Order of the President of the Russian Federation, 2021). The activities of the Fund are carried out using budgetary allocations from the federal government, voluntary contributions and donations, as well as other sources in accordance with the legislation of the Russian Federation. The funds are used to purchase medicine, equipment, rehabilitation devices and highly specialized surgeries.

**TABLE 3:** The supplementary list of life-threatening and chronic progressive rare diseases, leading to a reduction in life expectancy of citizens or their disability.

No	Name of the disease
1	Spinal muscular atrophy
2	Pompe disease
3	Familial Mediterranean fever
4	Cryopyrin-associated periodic syndrome
5	Tumor necrosis factor receptor-associated periodic syndrome
6	Hypophosphatasia
7	Mucopolysaccharidosis IV A
8	Neuroblastoma
9	Duchenne-Becker muscular dystrophy
10	Cystic fibrosis, according to approved categories
11	Short bowel syndrome
12	Tuberous sclerosis
13	Diabetes mellitus type 1 in children aged 0–4
14	Neuronal ceroid lipofuscinosis type II
15	Primary hyperoxaluria type I
16	Acute lymphoblastic leukemia
	Acute myeloblastic leukemia
	T-lymphoblastic lymphoma
	Primary immunodeficiency
	To provide medical care using the innovative method “Treatment of malignant diseases of blood and hematopoietic organs and severe nonmalignant blood diseases and congenital immunodeficiencies. These treatments include high-dose chemotherapy, transplantation of allogeneic TSRαβ-depleted hematopoietic progenitors and personalized therapy with genetically engineered drugs"
17	Epidermolysis bullosa
18	Lysosomal acid lipase deficiency
19	Hereditary retinal dystrophy caused by bi-allelic mutations in the RPE65 gene
	Types
	Leber congenital amaurosis (type II)
	Retinitis pigmentosa (type 20)
20	Congenital bile acid synthesis defect
	Children
	Children with clinical manifestations of cholestasis syndrome
	Confirmed congenital bile acid synthesis disorder using molecular genetic testing
21	Neurofibromatosis type 1
22	Hyper-IgD syndrome/mevalonate kinase deficiency
23	Urea Cycle Disorder
	Types:
	N-acetylglutamate synthetase (NAGS) deficiency;
	Carbamoyl phosphate synthetase 1 deficiency (CPSID)
	Ornithine Transcarbamylase Deficiency (Е72.4);
	Citrullinemia type 1
	Argininosuccinic aciduria;
	Argininemia
	Hyperornithinemia-hyperammonemia-homocitrullinuria (HHH) syndrome
	Citrullinemia type II
	Lysinuric protein intolerance
24	Lipodystrophy
25	Homozygous familial hypercholesterolemia
26	X-linked dominant hypophosphatemic rickets
27	NTRK fusion-positive cancers

Patients with rare diseases that are not included in the subsidized programs are not included in the registry. This fact significantly complicates the organization of medical care for these patients and makes budget planning exceptionally challenging. Therefore, it is extremely important to act on this issue and organize a systematic inclusion of new diseases in the subsidized lists and programs at both federal and regional levels.

## Epidemiological Analysis of Life-Threatening and Chronic, Progressive, Rare Diseases that Disable or Reduce the Life Expectancy

According to the Ministry of Health of the Russian Federation, the number of people included in “The regional list of patients with rare diseases” at the beginning of 2013 was 9,278 people including 4,962 children (53.5% of total). In 2018, this number had grown to 17,015 people including 8,639 children (50.8% of total). There was an 83% growth in the number of citizens included in the Federal Register from 2013 to 2018. The largest number of patients in the Register was with disorders of aromatic amino acid metabolism (28.5%) and idiopathic thrombocytopenic purpura (24.0%) (52.5% in total) (Table 4) (Accessibility of medical care and drug supply for patients with rare diseases in the Russian Federation: realities and ways of solving problems, 2015; Annual Bulletin of the Expert Council for rare (orphan) diseases, 2019).

**TABLE 4:** The distribution of patients from the “The regional list of patients with rare diseases” of the Registry as of January 1, 2018.

No	List of rare diseases	Proportion of all patients on the Registry (%)	Proportion of children in the Registry (%)	Prevalence^b^ per 100,000 people in Russia	Prevalence^c^ per 100,000 people in Europe
1	Hemolytic uremic syndrome^d^	2.4	67.0	0.28	0.1–0.9
2	Paroxysmal nocturnal hemoglobinuria (Marchiafava-Micheli syndrom)	2.1	3.1	0.26	1–9
3	Aplastic anemia, unspecified^d^	6.4	14.5	0.73	0.1–0.9
4	Inherited factor II deficiency (fibrinogen), factor VII deficiency (labile), factor X deficiency (Stewart-Prauer)^d^	1.3	38.3	0.15	0.1–0.9
5	Immune thrombocytopenia (Evans syndrome)	24.0	20.5	2.72	5–10
6	Complement deficiency	1.8	17.4		1–9
7	Central precocious puberty	5.5	97.5	0.64	0.8–3.2
8	Disorders of aromatic amino acid metabolism (classical phenylketonuria, other types of hyperphenylalaninemia)	28.5	77.0	3.25	5–10
9	Tyrosinemia^a^	<1	94.6	0.02	0.1–0.9
10	Maple syrup disease^a^	<1	95.5	0.01	0.1–0.9
11	Other types of amino acid metabolism disorders	<1	94.1	0.02	0.1–0.9
	Branched-chain amino acidemia (isovaleriс acidemia, methylmalonic acidemia, propionic acidemia)^a^				
12	Fatty acid metabolism disorders^a^	<1	72.3	0.04	1–9
13	Homocystinuria^a^	<1	75.9	0.02	1–9
14	Glutaric aciduria^a^	<1	91.1	0.04	0.1–0.9
15	Galactosemia	2,4	97.4	0.29	0.21
16	Other sphingolipidoses: Fabry disease, Niemann-Pick disease	<1	32.9	0.10	1–9
17	Mucopolysaccharidosis, type I^a^ ^,^ ^d^	<1	88.9	0.07	0.1–0.9
18	Mucopolysaccharidosis, type II^a^ ^,^ ^d^	<1	79.7	0.08	0.9–1.6
19	Mucopolysaccharidosis, type IV^a^ ^,^ ^d^	<1	62.3	0.04	0.1–0.9
20	Acute intermittent (hepatic) porphyria^a^	<1	12.0	0.07	1–5
21	Copper metabolism disorders (Wilson’s disease)	4.7	12.4	0.55	1–9
22	Osteogenesis imperfecta	4.2	55.1	0,50	5–10
23	Pulmonary (arterial) hypertension (idiopathic) (primary)	4.3	17.5	0.51	1–9
24	Systemic-onset juvenile rheumatoid arthritis^d^	8.2	74.7	0.96	1–9
Overall		100	50.8	11.6	–

a 11 diseases accounted for 4.2%.

b Prevalence rates per 100 thousand people in the Russian Federation as of January 01, 2018 for many diseases are significantly lower than epidemiological data found in scientific publications, studies, and reviews, both in Russia and in the world as a whole.

c Data on disease prevalence obtained from the European information system Orphanet (Orphanet Report Series, 2019).

d Drug supply for patients with these conditions is the responsibility of the federal executive body since 2020 (Angelis et al., 2015; Chiu et al., 2018).

As of January 1, 2018, on average, the number of patients with rare diseases from the “The regional list of patients with rare diseases” was 11.6 per 100,000 people with different dynamics in the regions of the Russian Federation (2015–2018).

Registering patients with rare diseases in the regions of the Russian Federation is influenced not just by objective factors associated with the problems of the healthcare system (lack of awareness of doctors about rare diseases, lack of diagnostic capabilities in the regions), but also by the readiness of regional authorities to finance drug supply for patients with rare diseases. Challenges with the willingness of pharmaceutical companies to work with rare diseases are also noted, along with the revealing of errors in statistical data.

## Disability Rate

The proportion of people with disabilities has increased from 51.7% in 2013 to 56.9% in 2018 among patients from “The regional list of patients with rare diseases” (Annual Bulletin of the Ex, 2019). The highest disability rate of patients was found in branched-chain amino acid metabolism disorders (95.0%), and the lowest disability rate was in patients with complement deficiency (24.9%). As of January 1, 2018, 42.4% of adults and 50.7% of children were registered with disabilities. The federal disability rate (adults and children cumulatively, who receive financial aid for treatment from the federal budget) was 80.7%.

## Case Fatality Rate

Comparison of case fatality rates among patients in 2013–2014 and in 2016–2017 showed that the absolute number of deaths increased 1.8-fold (from 175 to 315 people). Pediatric case fatality rates increased 1.4-fold (from 51 to 73 people), while the number of adults who died increased 1.95-fold (from 124 to 242 people).

Idiopathic thrombocytopenic purpura was the cause of the highest number of deaths in 2016–2017 (25.1% of all deaths in the “The regional list of patients with rare diseases”), pulmonary hypertension (22.5%) and unspecified aplastic anemia (21.0%, respectively). Compared to 2013–2014, the top three causes of death did not change. Among children, the proportion who died from pulmonary hypertension in 2016–2017 was 23.3%, while 9.6% of deaths were attributed to mucopolysaccharidosis type I 9.6%. Deaths from mucopolysaccharidosis type II, hemolytic-uremic syndrome, and aromatic amino acid metabolism disorders each contributed 8.2% to the total number of deaths.

The case fatality rates for different diseases from the “The regional list of patients with rare diseases” are presented in Table 5.

**TABLE 5:** The case fatality rates for different diseases from the “The regional list of patients with rare diseases” in 2013–2914 and 2016–2017.

No	List of rare diseases	Case fatality rates^a^ (adults + children)^b^ ^/^ ^c^, %	Case fatality rates (children)^b^ ^/^ ^c^, %	Case fatality rates (adults)^b^ ^/^ ^c^, %
1	Hemolytic uremic syndrome	4.76/2.78	3.18/2.46	12.5/3.45
2	Paroxysmal nocturnal hemoglobinuria (Marchiafava-Micheli syndrome)	5.65/3.11	−/−^d^	5.73/3.19
3	Aplastic anemia, unspecified	5.94/6.37	3.7/1.45	6.29/7.13
4	Inherited factor II deficiency (fibrinogen), factor VII deficiency (labile), factor X deficiency (Stewart-Prauer)	0/0.96	0/–^d^	0/1.53
5	Immune thrombocytopenia (Evans syndrome)	1.29/2.12	0.2/0.28	1.64/2.55
6	Complement deficiency	0/0.32	0/–^d^	0/0.39
7	Central precocious puberty	1.21/0.23	1.23/0.24	0/0
8	Disorders of aromatic amino acid metabolism (classical phenylketonuria, other types of hyperphenylalaninemia)	0.1/0.16	0.08/0.18	0.24/0.1
9	Tyrosinemia	0/6.45	0/6.90	0/0
10	Maple syrup urine disease	33.3/5.56	33.33/5.88	-/0^d^
11	Other types of amino acid metabolism disorders	0/3.23	0/3.45	0/0
	Branched-chain amino acidemia (isovaleriс acidemia, methylmalonic acidemia, propionic acidemia)			
12	Fatty acid metabolism disorders	26.46/1.85	24.14/2.56	40.0/0
13	Homocystinuria	0/0	0/-^d^	0/0
14	Glutaric aciduria	11.76/8.11	7.14/9.38	33.3/0
15	Galactosemia	0/0,78	0/0,80	0/0
16	Other sphingolipidoses: Fabry disease, Niemann-Pick disease	4.17/4.62	8.33/4.08	0/4.94
17	Mucopolysaccharidosis, type I	5.56/8.25	6.12/8.24	0/8.33
18	Mucopolysaccharidosis, type II	7.35/7.96	1.89/6.90	26.67/11.54
19	Mucopolysaccharidosis, type IV	2.63/6.25	3.7/10.34	0/0
20	Acute intermittent (hepatic) porphyria	0/4.17	−/−^d^	0/4.71
21	Copper metabolism disorders (Wilson’s disease)	0.9/1.49	0/1.01	1.02/1.56
22	Osteogenesis imperfecta	1.03/0.46	0.33/0.84	2.20/0
23	Pulmonary hypertension (idiopathic) (primary)	9.6/9.48	9.65/12.3	9.58/8.84
24	Systemic-onset juvenile rheumatoid arthritis	0.33/0.88	0.4/0.53	0/1.9
Overall		1.85/2.02	0.95/0.95	3.01/3.06

a Case fatality rate is an indicator equal to the ratio of the number of deaths from a certain disease in a certain period of time to the total number of people who had the same diagnosis within the same period of time, expressed as a percentage.

b Case fatality rates for the period 2013–2014/

c Case fatality rates for the period 2016–2017.

d No patients in the Registry.

It should be emphasized that a correlation between the number of deceased patients in the regions of the Russian Federation and lack or presence of the provision of appropriate medications for rare diseases to those in need has not yet been defined (Annual Bulletin of the Ex, 2019). We believe that such factors as the severity of the disease at the start of treatment, the presence of a medical care system for children with rare pathology in the region, including dynamic monitoring of the patient’s condition, can contribute to the case fatality rate.

## Medical Genetics Service

Medical genetics is an important resource for development of healthcare and its transition to personalized medicine, which is based on an individual approach to disease prevention and treatment. In 2018 the President of the Russian Federation, V.V. Putin, set a task for the Government of the Russian Federation to ensure accelerated development of genetic technologies, including the development of biological products, diagnostic systems, and immunobiological agents for healthcare. The Federal Scientific and Technical Program for the Development of Genetic Technologies in 2019–2027 (hereinafter referred to as the Program) and other national projects provide a comprehensive approach to addressing this task. The development and implementation of modern molecular genetic methods for predicting, diagnosing, and monitoring the course of diseases as well as methods of personalized pharmacotherapy are among the priority areas of the Strategy for Health Development in the Russian Federation up to the year 2025 (Decree of the President of the Russian Federation, 2019). Three centers in the format of consortia of leading genetic research organizations in Russia have been organized within the framework of the national project “Science” (2018–2024) and the Program to coordinate actions of the entire community of genetics scientists.

Medical care for patients with rare diseases is organized in accordance with the procedures and standards of medical care, as well as on the basis of clinical guidelines.

Medical care for patients with congenital and inherited diseases is provided in accordance with Order No. 917n of the Russian Ministry of Health of November 15, 2012. It covers the principles of medical care, genetic consultation office operating procedures, recommended staffing levels and the necessary equipment standards.

Standards of medical care are developed on the basis of clinical recommendations and include average indicators of the frequency of healthcare delivery and use of different medical therapies and services. These include average indicators for engagement with medical services, medications, use of medical devices including implantable devices, blood components, types of therapeutic nutrition, and other potential therapies based on the characteristics of the disease. The prescription and use of medicines, medical devices and specialized therapeutic foods that are not included in the relevant standard of medical care or not provided for by the relevant clinical recommendation are allowed if medically indicated, including in cases of severe, critical situations or if individuals are intolerant of standard therapies by decision of a medical commission. Standards of medical care for diseases from the “The regional list of patients with rare diseases” are available only for individual patient modes of care, which pertains to both primary and secondary healthcare in both pediatric and adult populations. Necessary standards were developed only in 38% of pediatric cases, 15% of adult cases and 6% of combined pediatric/adult cases. Standards were missing in 41% of cases for both children and adults.

In accordance with Federal Law No. 489-FL dated December 25, 2018, accessibility and quality of medical care are ensured not only by following procedures of medical care and standards of medical care, but also using clinical guidelines approved by specialized medical professional non-profit organizations. They are available for almost all rare diseases from the “The regional list of patients with rare diseases”.

**FIGURE 1:**
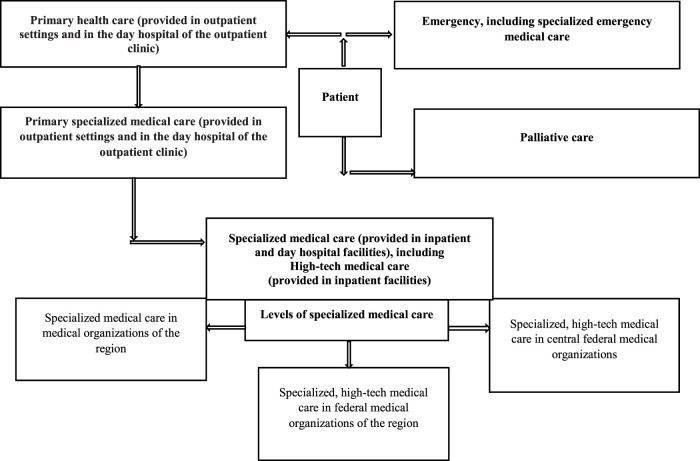
Principles of medical care for patients with rare diseases at the regional and federal levels in Russia.

Medical care for patients with rare diseases is provided free of charge in accordance with the state territorial guarantee program (Decree of the Government of the Russian Federation, 2020) (Figure 1).

In 2017 53.9% of patients from the “The regional list of patients with rare diseases” received specialized care in regional medical organizations (60.4% children and 46.8% adults), 6.3% in federal medical organizations located in their regions (7.4 and 5.0%, respectively), and 4.9% in federal medical organizations in other regions (8.2 and 1.3%). High-tech medical care was provided to 5.5% of patients (8.7 and 2.0%). It should be noted that only 10.4% of patients were referred to federal medical organizations, and 29.4% (15.3 and 44.9%) were not referred for specialized and high-tech medical care. It is important to emphasize that children were receiving all types of care more often than adults (84.7 vs 55.1%). A review of medical care delivery (secondary and highly specialized) to patients with rare diseases from “The regional list of patients with rare diseases” showed that there is almost no formal patient referral system in place (Komarov et al., 2019; Sokolov et al., 2019).

The principal responsibilities of genetics services are: medical-genetic counseling of families and patients with hereditary and congenital pathologies; the diagnostic process of hereditary diseases using cytogenetic, molecular-genetic and genetic-biochemical methods; mass screening of newborns for inherited metabolic disorders; selective screening of families and patients for inherited metabolic disorders; prenatal screening of pregnant women for common chromosomal abnormalities and congenital malformations; prenatal screening for common hereditary and congenital diseases; maintenance of a regional and federal register of families and patients with hereditary and congenital pathologies and monitoring their condition.

Preconception care is an effective and long-standing approach used in clinical genetics to reduce the genetic burden caused by genetic and chromosomal diseases. The possibility of its use is legislated by the Family Code of the Russian Federation (Article 15), as well as by Federal Law No. 323 from November 21, 2011 (Article 51) and it is free of charge at the place of residence (Federal Law No. (2021)).

**FIGURE 2:**
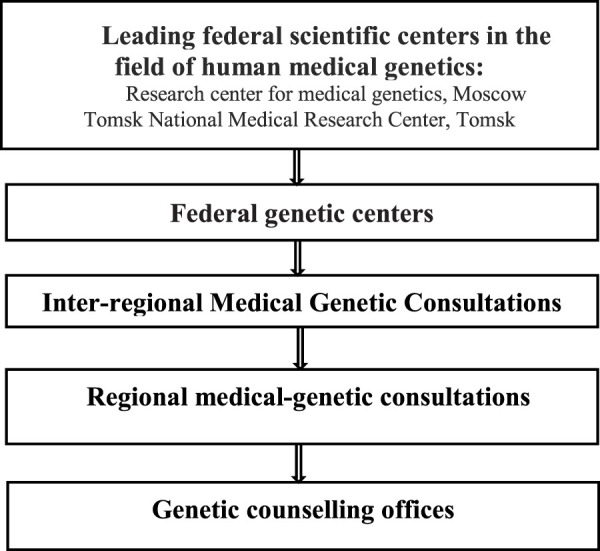
Medical genetics services in Russia.

The structure of the medical genetics service in Russia is shown in Figure 2.

Currently, 76 genetic consultation offices have been established and are functioning in 67 (out of 85) regions of the Russian Federation. There are only 340 medical geneticists and 395 genetic laboratory assistants in 79 regions of the Russian Federation that provide medical care in the field of medical genetics. At the same time, according to the regulatory documents, 3 full-time medical geneticists and 10 full-time genetic laboratory assistants are recommended per 1 million people (up to 10,000 births per year) to perform cytogenetic, molecular genetics and biochemical testing for prenatal, neonatal and selective screening.

Genetic consultation offices in 40 regions of the Russian Federation have molecular genetic laboratories, yet nevertheless, their number should be increased. In 39 regions genetic consultation offices have laboratories for selective screening for inherited metabolic disorders. There are also 84 cytogenetic laboratories performing prenatal and postnatal diagnosis of chromosomal abnormalities. Only 64% of genetic consultation offices are staffed with medical geneticists, while there is also a shortage of genetic laboratory assistants and bioinformaticians.

Identification of serum markers, expert-level fetal ultrasonography and risk calculation are carried out in medical genetic consultation offices (centers). These procedures are part of the prenatal screening of pregnant women for frequent chromosomal abnormalities and congenital fetal malformations. Screening measures are carried out in the first trimester of pregnancy in 80 regions of the Russian Federation. Thus, in 2019, 78.3% of pregnant women partook in prenatal screening.

**FIGURE 3:**
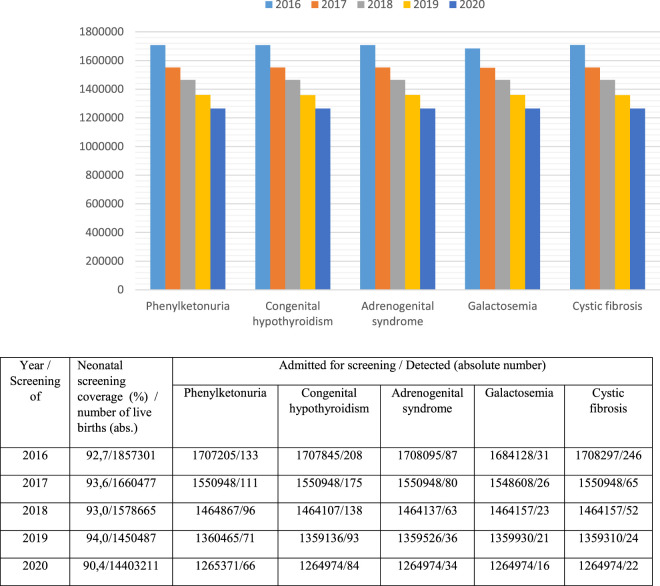
National neonatal screening coverage (2016–2020).

A program of newborn mass screening (neonatal screening) for five hereditary diseases has been implemented in 80 regions of Russia. Its purpose is early diagnosis, proper treatment and the prevention of disability and development of severe clinical consequences. It includes screening for phenylketonuria (since 1985), congenital hypothyroidism (since 1993), galactosemia (since 2006), cystic fibrosis (since 2006) and adrenogenital syndrome (since 2006). National neonatal screening coverage is over 95% (Figure 3), (Demographics of Russia explained, 2021).

As shown in Figure 3, from 2016 to 2020, there was a decrease in the absolute number of children admitted under the supervision of doctors of medical organizations (including for neonatal screening), which is primarily associated with a reduction in the childbirth rate. At the same time, the coverage of neonatal screening of children from 2016 to 2019 increased from 92,7 to 94,0%, but in 2020 it was only 90,4%. It can be assumed that the decrease in this index is associated with the unfavourable epidemiological situation for Covid-19 in many Russian regions. The lowest coverage with neonatal screening in 2020 was registered in the North Caucasian Federal District—80,0% and the Far Eastern Federal District—84,4%.

Two regions perform extended neonatal screening for inherited metabolic disorders using tandem mass spectrometry. 39 diseases are screened in the Primorsky region, while the Moscow region screens for 11 diseases. Currently, the issue of expanding neonatal screening to 39 diseases is being discussed. If it is used in all regions of Russia, up to 2,000 children can be diagnosed with inherited metabolic disorders. They can be treated with treatment regimens that have been developed based on diet-mediated therapies. Neonatal screening and initiation of therapy at the preclinical stage of the disease ensures high efficiency of treatment, increased survival rate and reduced children’s disability rate. Selective screening programs are also widely implemented.

In accordance with the Program of state guarantees of free medical care for 2021 through to 2023, prenatal testing, neonatal screening and medical genetic screening shall be carried out at the expense of budgetary allocations of the Regions of the Russian Federation in the appropriate clinical setting.

Full medical genetic care is provided by two federal centers - Research Center for medical genetics (Moscow) and the Tomsk National Research Medical Center of the Russian Academy of Sciences (Tomsk).

The creation of “Centers of expertise” for rare diseases contributes to solving the complex issues of establishing a rare diagnosis, selection and initiation of lifesaving treatment, and the follow-up of patients with rare diagnoses.

Supply of medications for patients with rare diseases in the Regions of the Russian Federation.

Problems relating to patient satisfaction with the supply of medications are addressed in the document “Strategy of drug supply for the population of the Russian Federation for the period until 2025 and the plan for its implementation” (hereinafter the Strategy). The principles of openness and awareness of State policy in the sphere of medication supply are implemented through monitoring the needs of medications of the population of the Russian Federation.

According to the data presented as of January 1, 2018, an average of 58.8% of patients in the regional segments of the Federal Register needed drug therapy, of which 91.3% were provided with the necessary medications. However, the presented figures varied significantly between different Regions of the Russian Federation.

The long-term objectives of the implementation of the Strategy for patients with rare diseases is to ensure equity in access to necessary medicines, including innovative ones, as well as specialized therapeutic foods, regardless of the region of residence. Only a federal program can guarantee a successful implementation of this strategy.

## Funding of the Preferential Drug Supply for Patients With Rare Diseases: Federal and Regional Levels

Expenditures on the subsidized supply of medications for patients from the “The regional list of patients with rare diseases” are taken from regional budgets and have increased almost fourfold over the period 2013–2018. In 2018 and 2019, the combined expenditures of the Regions of the Russian Federation (data from 68 Regions) for the subsidized supply of medications amounted to 13.2 and 11.6 billion rubles, respectively. The combined expenditures on the subsidized supply of medications for patients in the framework of “high-cost diseases program” noted in the federal budget for the same Regions were 12.0 and 20.8 billion rubles, respectively.

In 2020 the budget for rare diseases from the “The regional list of patients with rare diseases” was reduced to 10.1 billion rubles and then increased to 22.9 billion rubles in the framework of the “high-cost diseases” federal program. This was due to the fact that some diseases were transferred from regional funding to federal funding, which provided a more stable and guaranteed level of care for patients.

The treatment of each patient from the “The regional list of patients with rare diseases” in 2018 cost the regional budget on average 1 067 753 rubles, whereas the planned cost per patient included in the registry in 2020 was 807 600 rubles. An average patient of the “high-cost diseases” program in 2018 cost the federal budget 1,052,530 rubles, whereas the planned expenses in 2020 per patient included in the program registry were 1,813,274 rubles. (Krasilnikova and Smirnova, 2019).

## Opportunities of Compulsory Health Insurance in the System of Medical Care for Citizens With Rare Diseases

One of the options for potentially solving the problem of the lack of financial resources in regional budgets is utilizing the funds generated from compulsory health insurance payments. It could used to finance the supply of medications while providing medical care for patients from the “The regional list of patients with rare diseases”. The provision of vital and essential medicines to these patients is guaranteed in the Federal law from November 21, 2011 No 323-FL (article 80). (Federal Law No. (2021)). This falls under the remit of program of state guarantees for the delivery of primary care in the form of day hospital care, emergency care including specialized emergency care, palliative medical care and secondary care, including high-tech care (Decree of the Government, 2020). However, of note citizens with rare diseases who are being treated solely in outpatient settings are not guaranteed to have their pharmacological treatments covered by State funding. There are exceptions for certain categories of citizens who can be provided with measures of social support from the federal or regional budget, which also includes the provision of medicines for treatment in outpatient settings.

Access to medical care and drug supply for patients with rare diseases that are not included in the preferential state programs at the federal and regional level.

Information on the territorial representation of rare diseases, which are not included in the current subsidized programs of federal and regional levels in the regions of the Russian Federation, is noted to be presented with varying levels of accuracy. Commonly, there are clinical registers, which are formed and maintained by specialized federal institutions or NGOs.

The preferential supply of medications for patients with rare diseases, which are not included in the federal and regional programs, is made possible through use of the budgets of the Regions of the Russian Federation, provided that the disability status of a patient has been established. The Regions of the Russian Federation has the authority to form their own preferential lists of diseases and medicines. Also, charitable and non-budgetary funds can also engaged by patients to fund medical care (Krasilnikova and Smirnova, 2019).

## A Follow up of Patients With Rare Pathologies

The effectiveness of therapy and investment of financial resources in the treatment of patients with rare diseases should be evaluated by engaging a follow-up system. This is of critical importance given that 62.5% of regions have been shown to not have the presence of follow-up facilities for monitoring the health of these patients (Annual Bulletin of the Ex, 2019).

## Patients Organizations

Currently, in Russia there are dozens of non-profit public organizations (NGOs) that are divided into human rights organizations, patient support groups for those with rare diseases, funds and NGOs engaged mainly in charity as well as “umbrella” patient organizations uniting patients with different diseases and their families. The term “Public patient associations” is defined in the Federal law No. 323-FL from November 21, 2011 (Art. 28) (Federal Law No. (2021)). It describes the main operating rules of patient-orientated organizations. According to federal law, these organizations are allowed to participate in the development of norms and rules in the field of health care and to address questions related to violation of these rules.

These NGOs participate in the development and submission of proposals that help to improve regulatory frameworks that can lead to the protection of patients’ rights and the legitimate interests of patients. NGOs participate in the creation of modern mechanisms and procedures for interactions between patient associations and the government, medical organizations, businesses, international communities. They also draw the public’s attention to those problems of patients with rare diseases, as well as providing targeted financial and social support to patients (Abramov et al., 2018).

There are currently several special platforms for interaction between state authorities and public organizations: public councils on protection of patients’ rights under the Federal Health Care Oversight Service and its territorial branches; the Council of public organizations on the protection of patient’s rights under the Russian Ministry of Health; coordination councils on ensuring and protecting the rights of citizens in the compulsory health insurance system. Meanwhile, the State Duma Committee for Health Protection of Citizens with Rare Diseases expert Council was created in 2017.

## Discussion

It is extremely important to highlight the strengths and weaknesses of the organization of medical care for children with rare diseases in the Russian Federation.

The legal regulation of medical care for patients with rare diseases in Russia was established only 10 years ago, while it was established in the European Union (European Parliament and of the Council Regulation (EC), 2000) and the United States (Orphan drugs act of 1983, 1983) approximately 20 and 40 years ago, respectively. During this short period of time, a considerable amount of work has been completed. Firstly, a federal law was adopted, which provided normative and legal regulation in the sphere of health protection of citizens with rare diseases and guarantees of realization of these rights, as well as the creation of an official definition of rare diseases and associated medications were proposed. Further, the proposed management and referral systems for patients for receiving specialized and high-tech medical care was developed. Based on disease prevalence, a general list of rare diseases, regardless of the availability of relevant medications, consisted of 272 names of which 14 “high-cost diseases” were identified. The so-called Federal List of diseases was also established, the treatment of which is covered by the federal budget. At the same time, a “Regional List of Rare Diseases” consisting of 17 diseases was defined, the treatment of which is implemented at the expense of allocations from regional budgets. However, it can be seen that only 14 out of the 85 regions of Russia can be currently classified as performing “relatively well” in this domain. At the same time, some rare diseases on the “Regional List of Rare Diseases” have been transferred to the Federal List of Diseases, which has ensured stability for their future financing.

In 2021, a Presidential Decree was issued on the creation of a special Fund, providing allocations from the federal budget including voluntary property contributions and donations, to enable the provision of support for children with 27 other rare diseases.

It should be noted that with the improvement of diagnostics the number of citizens from the “The regional list of patients with rare diseases” has increased from 2013 to 2018 by 83.0%, totaling 21 594 by January 01, 2020.

A medical genetic service has been formed. In order to detect genetic diseases early, a program of mass screening of newborns for five hereditary diseases has been introduced in Russia. There is an ongoing discussion currently on the issue of the expansion of this program to encompass screening for 39 diseases in all regions of Russia.

A positive development is the growing number of patient organizations that unite patients with rare diseases and can influence the formation of policy in the field of rare diseases.

Despite positive developments, there is a persistent and substantial burden attributable to rare diseases. To date, Russia has not adopted a National Action Plan for patients with rare diseases. However, it is believed that perhaps multinational programs supported by general laws and cross-border agreements are likely to have a greater impact on issues faced by those patients with rare diseases than the individual programs of countries (Khosla and Valdez, 2018).

Numerous unresolved problems remain relating to the provision of drugs and specialized medical nutrition products for patients with rare diseases in several Regions of the Russian Federation. This is most evident when considering the availability of the provision of medications for patients not included in the State programs at both federal and regional levels. The shortage of regional budgetary funds makes it impossible to provide adequate drug supply to the above categories of patients. In this domain, it is not uncommon for the life and health of citizens in need of special treatment to be closely related to and even dependent upon the economic capabilities of a particular Region of the Russian Federation.

No scientific platform for the development and implementation of modern modes of diagnosis and treatment for hereditary diseases has been created. Domestic commercial production of drugs for rare diseases is underdeveloped due to a number of factors. Firstly, the low commercial incentives available to pharmaceutical companies to produce these medications and the subsequent lack of competition between manufacturers is exacerbated by the existing problem of import substitution. Further, small volumes of government procurement of drugs and government pricing policies which inevitably leads to price increases and the lack of an organizational structure for the development of these medications has further compounded matters.

There is a lack of knowledge and experience among doctors, primarily in primary care, in the early diagnosis, treatment and rehabilitation of patients with rare diseases. Issues concerning the transfer of adolescents with rare diseases from pediatric to adult settings are evidently apparent. In most Regions of the Russian Federation there are no functioning follow-up facilities for patients with rare diseases. This is therefore inhibiting physicians in acquiring and accumulating experience in managing these patients as well as patient care lacking interdisciplinary observation by specialists. Further, infrequent assessment of efficiency and quality of therapy is noteworthy, including those treatments of a high-tech nature or relating to rehabilitation. Of course, development and widespread implementation of technical support for registries of all patients with rare diseases, not only those receiving treatment, is also required.

It should be noted that there is a staff shortage of medical genetic specialists in a number of Regions, which leads to the limitation of timely diagnosis, treatment and prevention of genetic diseases. This could be contributing to why one third of patients with rare diseases are not referred to medical institutions for specialized or high-tech care. Nevertheless, the majority of children (84.7%) receive full, high-quality medical care.

Thus, the concept of the rarity of the disease should not mask the importance of existing problems. In our opinion, with limited funding, it is necessary to focus on those measures relating to the prevention of genetic diseases. A special role should be given not only to mass neonatal and selective screening, but also to screening prospective parents for the most frequent and severe mutations when they enter into legal marriage or when planning a pregnancy.

It is also important to emphasize that the use of proven methods based on evidence-based medicine with a positive therapeutic effect should be emphasized for those patients with rare diseases. Therefore, issues relating to indications for prescribing, contraindications and the cancellation of expensive drugs should be explored and established in the standards and clinical guidelines. Patients requiring ongoing monitoring, not only by regional specialists of the interdisciplinary team but also by specialists in reference centers (federal level centers) with knowledge and extensive experience in managing such patients, should be cared for while engaging greater use of telemedicine capabilities.

There is a need for changes in the organizational approach to the provision of specialized care, including high-tech care. Within the framework of programs of privileged medication provision, this will make it possible to promptly provide patients with rare diseases with necessary medications throughout the Russian Federation.

Important milestones for the improvement of medical care for the patients with rare diseases include: development of the Russian scientific platform; study of international experience and introduction of innovative technologies for the diagnostics and treatment of rare diseases; expansion of domestic production of drugs for rare diseases; centralized procurement and simplification of registration scheme in our country for new drugs produced in other countries; the training of highly qualified specialists including medical geneticists and doctors of other specialties. Solving many problems, including those relating to budgets and finances, in our opinion, is impossible without the joint activity of state authorities, compulsory medical insurance system, patient organizations and charitable foundations.

Finally, improving the quality of life of those families of children with rare diseases requires a comprehensive and strategic state policy, the improvement of mechanisms of medical care and social support, and the development of the system of social services, as well as changes in attitudes toward family values.

It also seems important to us to compare the approaches to patients with rare diseases between Russia and some European countries. Despite international initiatives for cooperation in the field of rare diseases, patient’s access to medications and medical services for rare diseases varies greatly from country to country. There are profound differences between countries in terms of the range and types of legislation, regulations and policies regarding rare disease medications (Gammie et al., 2015). When comparing access to these medications for rare diseases in Russia and some European countries, we can conclude that in such countries as the United Kingdom, France, Germany, Italy and Spain, more effective solutions are in existence. They were most accessible to all patients in Germany and France. In other European countries, 30–60% of the money spent on the purchase of medications for rare diseases is reimbursed (Package Management Orphan Drugs, 2017; Zamora et al., 2019).

Some countries, such as France, have introduced the flexibility to regulate and use imported drugs for indications that are not registered in the country. It is also proposed to introduce Early Access Programs, which will alleviate the urgent medical need for those medications for rare diseases (Czech et al., 2020).

Within the State program of Russia, 2 programs of preferential (free for patients) provision of patients with rare diseases from federal (14 diseases) and regional (17 diseases) budgets are guaranteed. Further, coverage from the Presidential Fund (27 diseases) has also been adopted, which will expand the possibilities of providing patients with rare disease medications. The use of those medications not registered in Russia for the treatment of rare diseases remains a persistent problem.

Many European countries pay great attention to neonatal screening: studies for the largest number of rare diseases are conducted in Poland (Regulation (EC), 2019) and the Netherlands (Young et al., 2017). In Russia, mass screening of newborns is performed for just five diseases, but its expansion is being discussed.

In order to develop a common Rare Disease Action Strategy, the European Council recommended in 2009 that EU member states develop and adopt a National Action Plan to ensure effective and efficient recognition, prevention, diagnosis, treatment, care and research of rare diseases in Europe (Commission of the European Communities, 2008; Rodwell and Aymé, 2015). Currently, Russia is also discussing the formation of a Strategy for Rare Diseases.

A major concern of many countries is the formation of rare disease registries, which are public, private non-profit or for-profit entities (Boulanger et al., 2020; Rare Disease Registries in Europe, 2020). Russia maintains a federal register and its regional segments is only for patients who receive drug therapy. Information on other diseases can only be obtained from patient organizations, but its reliability is questionable.

## Conclusion

Over the past decade, Russia has achieved some success in the field of rare disease policy. Legal and regulatory frameworks of medical care have been formed, while patients now receive specialised, modern, and highly advanced treatment. Regional and federal programmes of preferential (free) drug coverage for patients (58 diseases) have also been introduced.

At the same time, some challenges remain unresolved, including the lack of timely diagnostic services relating to rare diseases and choices relating to treatment plans. The organisation of patient’s follow-up and rehabilitation services are also lacking. It is worth mentioning that the absence of a National Action Plan for Rare Diseases in Russia reflects the lack of government interest in expanding research in the field of genetic diseases. Another significant challenge for healthcare systems in Russia is the financing of treatment. It includes the high prices of medications, the purchasing of which carries out with prices 2–3 times higher than in Europe; conducting clinical trials for new medicines; the extension of mass screening of newborns; prompt provision and redistribution of orphan medications between regions as well as the production of drugs domestically.

Given the associated high costs of treatment for rare diseases, more cost-effective interventions should be engaged to allocate resources better and find ways to reduce costs to society and individual patients. An emphasis should be placed on developing and promoting preventive programs, evaluating the effectiveness of treatments, and patient’s quality of life.

It is also critically important to study the innovative experience of European countries, the United States, Japan in the field of organizing medical and psycho-social health services to people with rare diseases, participation in the creation of an international register of rare diseases, allowing getting exclusive data related to the effectiveness of performed treatment and results of innovative technology introduction into the diagnosis of orphan diseases, novel therapy methods and rehabilitation of these patients.
